# Spread of Chikungunya Virus East/Central/South African Genotype in Northeast Brazil

**DOI:** 10.3201/eid2310.170307

**Published:** 2017-10

**Authors:** Antonio Charlys da Costa, Julien Thézé, Shirley Cavalcante Vasconcelos Komninakis, Rodrigo Lopes Sanz-Duro, Marta Rejane Lemos Felinto, Lúcia Cristina Corrêa Moura, Ivoneide Moreira de Oliveira Barroso, Lucineide Eliziario Correia Santos, Mardjane Alves de Lemos Nunes, Adriana Avila Moura, José Lourenço, Xutao Deng, Eric L. Delwart, Maria Raquel dos Anjos Silva Guimarães, Oliver G. Pybus, Ester C. Sabino, Nuno R. Faria

**Affiliations:** University of São Paulo, São Paulo, Brazil (A.C. da Costa, E.C. Sabino);; University of Oxford, Oxford, United Kingdom (J. Thézé, J. Lourenço, O.G. Pybus, N.R. Faria);; School of Medicine of ABC (FMABC), Santo Andre, Brazil (S.C.V. Komninakis);; Federal University of São Paulo, São Paulo (S.C.V. Komninakis, R.L. Sanz-Duro);; Laboratório Central de Saúde Pública Dra. Telma Lobo, Secretaria de Estado da Saúde da Paraíba, João Pessoa, Brazil (M.R.L. Felinto, L.C.C. Moura);; Hospital Unimed Maceió, Alagoas, Brazil (I.M. de Oliveira Barroso, L.E.C. Santos, M.R. dos Anjos Silva Guimarães);; Hospital Escola Dr. Helvio Auto, Alagoas (M.A. de Lemos Nunes, A.A. Moura);; Universidade Federal de Alagoas, Maceió, Brazil (A.A. Moura);; Blood Systems Research Institute, San Francisco, California, USA (X. Deng, E.L. Delwart);; University of California, San Francisco, California, USA (X. Deng, E.L. Delwart)

**Keywords:** chikungunya virus, ECSA genotype, Maceió, genetic epidemiology, Zika virus, dengue virus, Brazil, East/Central/South African genotype, viruses, vector-borne infections

## Abstract

We investigated an outbreak of exanthematous illness in Maceió by using molecular surveillance; 76% of samples tested positive for chikungunya virus. Genetic analysis of 23 newly generated genomes identified the East/Central/South African genotype, suggesting that this lineage has persisted since mid-2014 in Brazil and may spread in the Americas and beyond.

Dengue virus (DENV), Zika virus (ZIKV), and chikungunya virus (CHIKV) co-circulate in Brazil, are predominantly transmitted by *Aedes aegypti* mosquitoes, and cause similar clinical symptoms upon infection, complicating epidemiologic surveillance. Brazil harbors the highest diversity of CHIKV in the Americas; both the Asian and the East/Central/South African (ECSA) lineages circulate in the country (*1*). Despite high prevalence of CHIKV in Brazil (352,773 notified cases during January 2016–May 2017) and its widespread distribution (*2*), little is known about its transmission. We report a molecular and genomic investigation of an outbreak of CHIKV infection in Maceió, Alagoas state, Northeast Brazil.

During March 30–May 3, 2016, ≈12,000 patients visited 2 private hospitals in Maceió; roughly 70% of them had exanthematous illness symptoms compatible with DENV, CHIKV, or Zika virus infection. We analyzed 273 randomly chosen samples by using molecular diagnostics and virus discovery methods. The study was approved by the Faculty of Medicine from the University of São Paulo Review Board, and we obtained informed consent from all participants. 

Analyzed samples were from patients who were on average 37 years of age (range 1–86 years); 175 (64%) were female, and 198 (73%) resided in Maceió municipality. Diagnostic tests for DENV, ZIKV, and CHIKV confirmed that 208 (76%) were positive for CHIKV RNA (Technical Appendix). In addition, 66 (24%) were positive for Zika virus RNA and 36 (13.2%) were co-infected with CHIKV and Zika virus, consistent with Zika virus circulation in Northeast Brazil in mid-2016 (*3*). We detected no DENV infections. Cycle threshold (C_t_) values for CHIKV RNA–positive samples were lower (average C_t_ = 24.6) than those for ZIKV (average C_t_ = 33.5).

We applied a metagenomics next-generation sequencing protocol to 38 randomly chosen CHIKV RNA–positive samples (*4*) (Technical Appendix). We recovered 23 CHIKV genomes (>4,000 bp) by using the MiSeq Sequencer (Illumina, Inc., San Diego, CA, USA); mean genome coverage was 72× and mean depth coverage 207× (Technical Appendix Table 1). We also included 2 CHIKV RNA–positive samples from João Pessoa, Paraíba state. We did not detect the E1-A226V adaptive mutation associated with large outbreaks in Asia (*5*) in the strains we analyzed. We then appended sequences to publicly available data (659 CHIKV isolates) and used maximum-likelihood and Bayesian phylogenetic analysis to identify the origins of the outbreak (Technical Appendix).

On the basis of available sequences of isolates from the Americas, the Maceió sequences we analyzed fell within a single strongly supported monophyletic clade (bootstrap support = 99%, posterior support = 1.00) that belongs to the ECSA genotype (Figure). Genetic analysis suggests the outbreak most likely originated from transmission cycles not previously identified in Northeast Brazil and not from a separate introduction into the Americas. Before August 2015, no CHIKV infections had been reported in Alagoas (Figure). Molecular dating analysis indicates that the outbreak was caused by a single founder strain that is estimated to have arrived in Alagoas around late April 2015 (95% Bayesian credible interval July 2014–October 2015), possibly a few months before the earliest reports of CHIKV there (Figure). Our reconstruction of the history of the ECSA genotype in Brazil using a phylogeographic approach (*6*) further suggests that this lineage was introduced into Alagoas from the neighboring Bahia state, which experienced a CHIKV epidemic during January–August 2015 (*7*).

**Figure:**
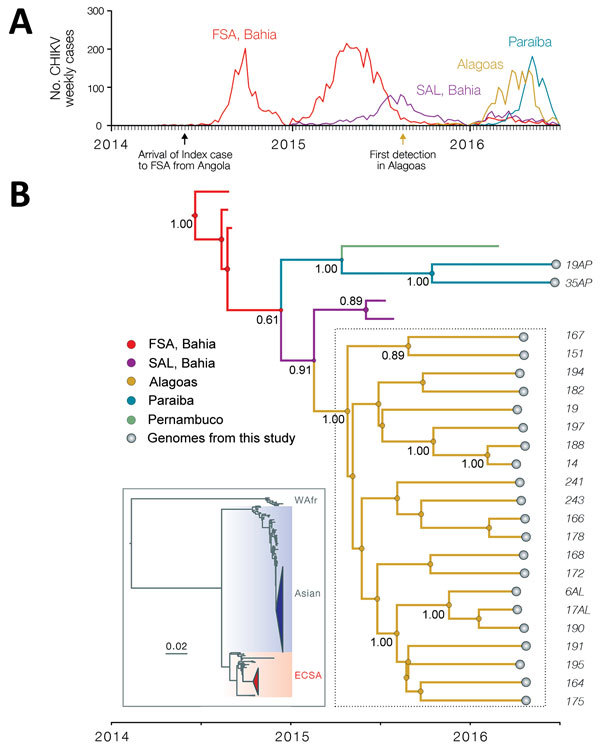
Epidemiologic and genetic surveillance of CHIKV in Northeast Brazil. A) Notified CHIKV cases for Alagoas state (Maceió municipality), Paraíba state (João Pessoa municipality), and Feira de Santana (FSA) and Salvador (SAL) municipalities (*3*), both located in the Bahia state. B) Molecular clock phylogeny obtained using 23 novel CHIKV sequences (with length >4,000 nt) collected in Northeast Brazil (dashed box). Numbers along branches represent clade posterior probability >0.75. Colors in branches represent most probable locations. At each node, size of the colored circles is proportional to location posterior probability. Inset shows a maximum-likelihood phylogeny with all publicly available CHIKV genome sequences (n = 659). The Indian Ocean Lineage (IOL) genotype has been collapsed. Triangles represent clades circulating in the Americas; the American-ECSA lineage reported in this study is shown in red and the American-Asian lineage in blue. CHIKV, chikungunya virus; ECSA, East/Central/South African genotype; WAfr, West African genotype.

The brief to negligible period of undetected transmission of CHIKV in Alagoas is consistent with past reports (*8*) and in contrast with the unrecognized circulation in the region of Zika virus, which typically causes milder symptoms (*3*). The most common clinical signs and symptoms for the sequenced CHIKV cases were fever (87%), arthralgia (70%), headache (44%), exanthema (30%), and myalgia (26%) (Technical Appendix Table 2). CHIKV infection is often characterized by prolonged periods of disability. Further investigation is needed to study potential differences in the effects of CHIKV and Zika virus infection on public health, as well in pathology and innate and adaptive immune responses to each genotype.

The unrecognized transmission of the CHIKV ECSA genotype in Northeast Brazil is unique in the Americas. The spread of this genotype in this region will be mediated by several factors, including herd immunity, vector suitability, and human mobility. Serologic and molecular surveys in human and mosquito populations are required to characterize the factors involved in transmission and the extent of cross-protection of the Asian and the ECSA genotypes in the Americas. Although CHIKV ECSA has been found only in *Ae. aegypti* mosquitoes (*9*), a recent study has shown that *Ae. albopictus* mosquitoes in Brazil are also highly competent in CHIKV ECSA transmission (*10*). Given the widespread distribution of both vectors in the Americas (*1*), it is possible that the ECSA lineage may spread to other regions in the Americas and beyond. A better understanding of the transmission dynamics of CHIKV, DENV, and Zika virus in the Americas is essential to fully understand the risk of arbovirus-associated congenital anomalies, Guillain-Barré, and other neurological syndromes.

Technical Appendix Additional information about the testing, sequencing, and analysis of chikungunya virus genomes.
