# Supplementary material for: Spread of Chikungunya Virus East/Central/South African Genotype in Northeast Brazil
Source: Emerg Infect Dis. 2017 Oct;23(10):1742–4. doi: 10.3201/eid2310.170307 (PMC5621546; doi:10.3201/eid2310.170307)
Supplement: Technical Appendix — Additional information about the testing, sequencing, and analysis of chikungunya virus genomes. [file 17-0307-Techapp-s1.pdf]

Article DOI: https://doi.org/10.3201/eid2310.170307

Spread of Chikungunya Virus East/Central/South African Genotype in Northeast Brazil

Technical Appendix

Laboratory testing

Viral RNA was extracted from 140 µL of serum samples using QIAmp viral RNA kit (QIAGEN, Valencia, CA, USA) and RT-qPCR analyses were performed for DENV serotypes 1–4 (*1*), ZIKV (*2*) and CHIKV (*3*). Of 273 samples tested, 76% (n = 208) were CHIKV-RNA$^+$, 24% (n = 66) were ZIKV-RNA$^+$ and 13.2% (n = 36/273) were co-infected with CHIKV and ZIKV, similar to recent findings from Salvador, Bahia (*4*).

Metagenomic sequencing

To identify the cause of the outbreak, each plasma sample was subjected to centrifugation at 15,000xg for 10 minutes, filtered through a 0.45 µm filter (Merck Millipore, Billerica, MA, USA). The filtrates were treated with a mixture of nuclease enzymes to digest unprotected nucleic acids. Viral nucleic acids were extracted using Maxwell 16 Viral Total Nucleic Acid Purification Kit (Promega, Inc., Madison, WI, USA) and cDNA synthesis was performed with AMV Reverse transcription (Promega, Inc., Madison, WI, USA). A second strand of cDNA synthesis was performed using DNA Polymerase I Large (Klenow) Fragment (Promega, Inc., Madison, WI, USA). Subsequently, a Nextera XT Sample Preparation Kit (Illumina, Inc., San Diego, CA, USA) was used construct a DNA library, with each sample identifiable using dual barcodes. For size selection, we used a Pippin Prep (Sage Science, Inc.) to select a 400bp insert (range 200–600bp). The library was deep-sequenced using the MiSeq Sequencer (Illumina, Inc., San Diego, CA, USA) with 300 bped ends. BLASTx was used to identify viral sequences through their protein sequence similarity to annotated viral proteins in GenBank search, as

previously described (*5*). Of the 40 samples that were tested, we were able to obtain 23 complete or near-complete genomes (>4000bp; Genbank Accession numbers KY704933 to KY704955).

Phylogenetic analysis

Publicly available chikungunya virus genome data (>1500bp) was retrieved from Genbank on 17 Feb 2017 using an in-house script. Newly generated genomes (>4000bp) were then appended to the publicly available data and aligned using MAFFT (*6*). A maximum likelihood phylogeny was constructed for the collated dataset (n = 659) using PhyML with 500 bootstrap replicates (*7*). The correlation between genetic divergence and collection dates for the ECSA genotype subtree ($r^2 = 0.41$, rate = 8.9×10^{-4} substitutions per site per year) was inspected using TemPest (*8*). Dated phylogenies were constructed using a Bayesian framework implemented in the BEAST software package (*9*).

To reconstruct date phylogenies, we used a SDR06 nt substitution model with a non-informative Bayesian Skygrid coalescent tree prior (20 grid points) (*10*) and a strict molecular clock model. Finally, a discrete phylogeographic analysis using an asymmetric substitution model (*11*) was conducted to investigate the origins and spread across considered locations (n = 3 from Feira de Santana, Bahia, n = 1 from Pernambuco, n = 2 from João Pessoa, Paraíba, n = 2 from Salvador, Bahia, n = 21 from Maceió/Capelas, Alagoas).

References

1. Lai YL, Chung YK, Tan HC, Yap HF, Yap G, Ooi EE, et al. Cost-effective real-time reverse transcriptase PCR (RT-PCR) to screen for Dengue virus followed by rapid single-tube multiplex RT-PCR for serotyping of the virus. J Clin Microbiol. 2007;45:935–41. PubMed http://dx.doi.org/10.1128/JCM.01258-06

2. Lanciotti RS, Kosoy OL, Laven JJ, Velez JO, Lambert AJ, Johnson AJ, et al. Genetic and serologic properties of Zika virus associated with an epidemic, Yap state, Micronesia, 2007. Emerg Infect Dis. 2008;14:1232–9. PubMed http://dx.doi.org/10.3201/eid1408.080287

3. Lanciotti RS, Kosoy OL, Laven JJ, Panella AJ, Velez JO, Lambert AJ, et al. Chikungunya virus in US travelers returning from India, 2006. Emerg Infect Dis. 2007;13:764–7. PubMed http://dx.doi.org/10.3201/eid1305.070015

4. Sardi SI, Somasekar S, Naccache SN, Bandeira AC, Tauro LB, Campos GS, et al. Coinfections of Zika and chikungunya viruses in Bahia, Brazil, identified by metagenomic next-generation sequencing. J Clin Microbiol. 2016;54:2348–53. PubMed http://dx.doi.org/10.1128/JCM.00877-16

5. Deng X, Naccache SN, Ng T, Federman S, Li L, Chiu CY, et al. An ensemble strategy that significantly improves de novo assembly of microbial genomes from metagenomic next-generation sequencing data. Nucleic Acids Res. 2015;43:e46. PubMed http://dx.doi.org/10.1093/nar/gkv002

6. Katoh K, Standley DM. MAFFT multiple sequence alignment software version 7: improvements in performance and usability. Mol Biol Evol. 2013;30:772–80. PubMed http://dx.doi.org/10.1093/molbev/mst010

7. Guindon S, Dufayard JF, Lefort V, Anisimova M, Hordijk W, Gascuel O. New algorithms and methods to estimate maximum-likelihood phylogenies: assessing the performance of PhyML 3.0. Syst Biol. 2010;59:307–21. PubMed http://dx.doi.org/10.1093/sysbio/syq010

8. Rambaut A, Lam T, de Carvalho L, Pybus OG, Exploring the temporal structure of heterochronous using TemPest, Virus Evolution 2:vew007.

9. Drummond AJ, Suchard MA, Xie D, Rambaut A. Bayesian phylogenetics with BEAUti and the BEAST 1.7. Mol Biol Evol. 2012;29:1969–73. PubMed http://dx.doi.org/10.1093/molbev/mss075

10. Gill MS, Lemey P, Faria NR, Rambaut A, Shapiro B, Suchard MA. Improving Bayesian population dynamics inference: a coalescent-based model for multiple loci. Mol Biol Evol. 2013;30:713–24. PubMed http://dx.doi.org/10.1093/molbev/mss265

11. Lemey P, Rambaut A, Drummond AJ, Suchard MA. Bayesian phylogeography finds its roots. PLOS Comput Biol. 2009;5:e1000520. PubMed http://dx.doi.org/10.1371/journal.pcbi.1000520

Technical Appendix Table 1. Sequencing details of the chikungunya virus isolates reported in this study

Accession number	Isolate name	No. reads mapped	Mean depth coverage	Genome coverage (%)*
KY704933	6AL	19,267	175.79	79.02
KY704934	14	5,221	54.18	62.27
KY704935	17AL	2,847	44.05	73.63
KY704936	19	11,517	53.39	61.72
KY704937	151	3,064	47.40	48.03
KY704938	164	1,010	16.90	67.16
KY704939	166	36,801	467.31	94.97
KY704940	167	1,992	32.16	72.51
KY704941	168	796	16.79	52.21
KY704942	172	18,577	249.79	95.53
KY704943	175	8,561	82.14	78.84
KY704944	178	1,004	21.48	60.91

Accession number	Isolate name	No. reads mapped	Mean depth coverage	Genome coverage (%)*
KY704945	182	74,785	328.01	51.82
KY704946	188	6,927	77.66	66.64
KY704947	190	9,650	146.31	84.09
KY704948	191	13,041	62.36	56.36
KY704949	194	44,321	335.58	59.84
KY704950	195	16,140	168.99	67.76
KY704951	197	650	16.38	58.19
KY704952	241	1,192,684	2,050.39	93.76
KY704953	243	3,342	50.44	74.80
KY704954	19AP	15,832	151.30	100.00
KY704955	35AP	11,206	111.64	100.00

*Relative to reference genome sequence KU940225.

Technical Appendix Table 2. Clinical information of sequenced chikungunya virus isolates*

Isolate	CT	Sex, age (y)	Date	Location	Clinical symptoms
6AL	24.9	F, 44	30–03–16	Santa Lúcia, Maceió, AL	Fever, arthralgia
14	21.4	M, 30	30–03–16	Poço, Maceió, AL	Fever, arthralgia, muscle pain, headache
17AL	21.7	F, 50	01–04–16	Centro, Maceió, AL	Fever, muscle pain, nausea
19	19.5	F, 31	01–04–16	Santos, Maceió, AL	Fever, arthralgia, myalgia, fatigue, exanthema
151	35.8	M, 64	15–04–16	Tab. do Pinto, Maceió, AL	Fever, arthralgia, edema
164	33.9	F, 25	19–04–16	Clima Bom, Maceió, AL	Fever, arthralgia, headache
166	29.5	M, 34	17–04–16	Poço, Maceió, AL	Fever, arthralgia, headache
167	28.2	M, 30	14–04–16	Vergel, Maceió, AL	Fever, arthralgia, muscle pain
168	36.9	F, 43	14–04–16	Vergel, Maceió, AL	Fever, arthralgia, headache
172	18.2	M, 18	16–04–16	FernãoVelho, Maceió, AL	Fever, arthralgia, headache
175	19.5	F, 28	19–04–16	Poço, Maceió, AL	Arthralgia, exanthema
178	20.2	M, 41	19–04–16	Centro, Maceió, AL	Fever, pruritus
182	19.3	F, 17	14–04–16	Santa Amélia, Maceió, AL	Fever, arthralgia, maculopapular rash
188	25.8	F, 30	17–04–16	Centro, Capela, AL	Fever, arthralgia, headache, nausea
190	36.0	F, 61	15–04–16	Mangabeiras, Maceió, AL	Fever, arthralgia, myalgia, headache, retro-orbital pain
191	28.8	F, 36	19–04–16	Farol, Maceió, AL	Fever, headache, myalgia, arthralgia, leukopenia
194	23.3	F, 60	07–04–16	Pajuçara, Maceió, AL	Fever, arthralgia, myalgia, fatigue
195	17.6	F, 39	07–04–16	Poço, Maceió, AL	Fever, arthralgia, myalgia, fatigue
197	21.8	F, 21	13–04–16	Farol, Maceió, AL	Fever, myalgia, arthralgia
241	19.8	M, 11	07–04–16	Centro, Maceió, AL	Fever, headache, maculopapular rash, vomiting
243	26.2	F, 41	09–04–16	Sta. Amélia, Maceió, AL	Muscle pain, skin rash
19AP	16.5	F, 20	20–06–16	Centro, João Pessoa, PB	Vomiting, Fever, Headache, maculopapular rash
35AP	26.4	F, 32	17–06–16	Bayeux, João Pessoa, PB	Muscle pain, maculopapular rash

*Location indicates neighborhood, municipality and federal state (AL = Alagoas, PB = Paraíba). Date of sample collection is shown as dd-mm-yy. CT = qPCR-CT value.
